# Activation of the autophagy pathway decreases dengue virus infection in *Aedes aegypti* cells

**DOI:** 10.1186/s13071-021-05066-w

**Published:** 2021-10-26

**Authors:** Tse-Yu Chen, Chelsea T. Smartt

**Affiliations:** Florida Medical Entomology Laboratory, Department of Entomology and Nematology, University of Florida, Vero Beach, FL USA

**Keywords:** Autophagy, Dengue virus, *Aedes aegypti*, Aag-2 cells

## Abstract

**Background:**

Mosquito-borne dengue virus (DENV) causes major disease worldwide, impacting 50–100 million people every year, and is spread by the major mosquito vector *Aedes aegypti*. Understanding mosquito physiology, including antiviral mechanisms, and developing new control strategies have become an important step towards the elimination of DENV disease. In the study reported here, we focused on autophagy, a pathway suggested as having a positive influence on virus replication in humans, as a potential antiviral target in the mosquito.

**Methods:**

To understand the role played by autophagy in *Ae. aegypti*, we examined the activation of this pathway in Aag-2 cells, an *Ae. aegypti*-derived cell line, infected with DENV. Rapamycin and 3-methyladenine, two small molecules that have been shown to affect the function of the autophagy pathway, were used to activate or suppress, respectively, the autophagy pathway.

**Results:**

At 1-day post-DENV infection in Aag-2 cells, transcript levels of both the microtubule-associated protein light chain 3-phosphatidylethanolamine conjugate (LC3-II) and autophagy-related protein 1 (ATG1) increased. Rapamycin treatment activated the autophagy pathway as early as 1-h post-treatment, and the virus titer had decreased in the Aag-2 cells at 2 days post-infection; in contrast, the 3-methyladenine treatment did not significantly affect the DENV titer. Treatment with these small molecules also impacted the ATG12 transcript levels in DENV-infected cells.

**Conclusions:**

Our studies revealed that activation of the autophagy pathway through rapamycin treatment altered DENV infection in the mosquito cells, suggesting that this pathway could be a possible antiviral mechanism in the mosquito system. Here we provide fundamental information needed to proceed with future experiments and to improve our understanding of the mosquito’s immune response against DENV.

**Graphical Abstract:**

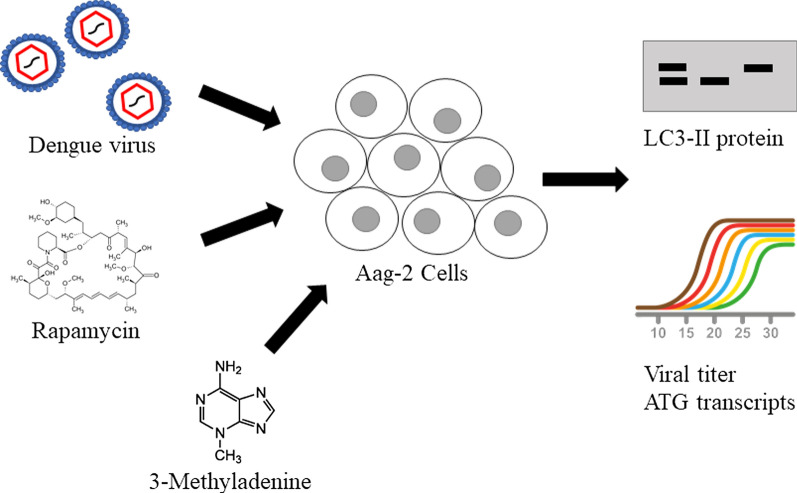

**Supplementary Information:**

The online version contains supplementary material available at 10.1186/s13071-021-05066-w.

## Background

Dengue virus (DENV) is a single-strand RNA virus with four serotypes, DENV-1, DENV-2, DENV-3 and DENV-4. One disease caused by DENV is dengue fever and because of antibody-dependent enhancement, where the antibody against the first serotype cannot neutralize the virus particles of a different serotype [1], some cases will turn into severe dengue fever. According to the World Health Organization, 5.2 million dengue cases were reported in 2019 and the number of dengue cases increased eightfold in the past two decades [2]. Moreover, one study estimated 3.9 billion people in 128 countries are at risk of DENV disease [3].

Autophagy is a pathway with a role in maintaining cellular homeostasis, including the removal of unnecessary or dysfunctional components in the cell, facilitated by several autophagy-related (ATG) proteins, and controls extensive cellular remodeling [4]. ATG proteins are required for autophagosome formation. Additional roles of autophagy in cells are to balance sources of energy and to respond to stress [5]. Autophagy is also involved in removing misfolded or aggregated proteins [6], clearing damaged organelles [7, 8] and eliminating intracellular pathogens [9, 10]. Failure in the autophagy function to maintain homeostasis is likely to trigger cell death [11]. The involvement of this pathway can be determined by the detection of microtubule-associated protein light chain 3 (LC3) protein. Increased LC3-II (microtubule-associated protein light chain 3-phosphatidylethanolamine conjugate) levels can be associated with autophagosome synthesis [12, 13]. After the maturation of the autophagosome, it fuses with the lysosome, forming the autolysosome, to degrade the contents inside [14]. Autophagy has also been shown to be involved in immunity where pattern recognition receptors will trigger autophagy to eliminate intracellular microorganisms in humans [15, 16] and *Drosophila* [16–19].

DENV infection in humans has been shown to trigger the autophagy pathway [20], and this activation enhances DENV replication [21]. Studies in mammalian cell lines revealed that DENV nonstructural protein NS4A was able to induce autophagy and protect cells from death during infection [22]. Moreover, DENV-induced autophagy resulted in the release of free fatty acids that increased the efficiency of virus replication [23]. Another DENV nonstructural protein, NS1, can also increase LC3-II protein levels as well as cause vascular leakage [24]. The endoplasmic reticulum stress induced by DENV is required for autophagy activation and contributes to viral replication [25].

Unlike the mammalian system, in *Drosophila* the autophagy pathway has been shown to target intracellular pathogens [26]; however, the interaction between autophagy and virus is not clear in the mosquito. Although it has been reported that in DENV-2 infected *Ae. aegypti* the autophagy-related genes had higher expression levels and autophagosomes were detected in the midgut [27], the role of the autophagy pathway as an antiviral or proviral mechanism in the mosquito system is still unidentified.

Here we investigated the role of the autophagy pathway in an *Ae. aegypti* cell line Aag-2. The small molecules rapamycin and 3-methyladenine (3-MA) were used to manipulate the autophagy pathway, as an activator and an inhibitor, respectively. Rapamycin is an inhibitor of the mammalian target of rapamycin complex 1 (mTORC1) [28] and, therefore, can be used as an inducer of autophagy [29]. Moreover, the mTORC1-derived inhibition of the autophagy pathway is crucial during DENV infection [30]. On the other hand, 3-MA is a specific inhibitor of phosphoinositide 3-kinase (PI3K) and is known to interfere with autophagosome formation [31]. We observed that LC3-II levels increased in the Aag-2 cells after DENV-2 infection. The treatment with rapamycin significantly decreased the viral titer in Aag-2 cells while the 3-MA treatment did not. Taken together, our results indicate that autophagy might be involved as an antiviral mechanism in the mosquito and are an important step to understanding mosquito immunity.

## Methods

### Cell culture

Aag-2 cells were cultured in Schneider’s Drosophila Media (Gibco, Thermo Fisher Scientific, Waltham, MA, USA) supplemented with 10% fetal bovine serum and 1% Antibiotic/Antimycotic Solution (Gibco, Thermo Fisher Scientific) in an incubator at 32 °C.

### Small molecule and virus infection in Aag-2 cells

Aag-2 cells were seeded into 6-well plates and incubated overnight to reach 80% confluency. Rapamycin (Ubiquitin; Proteasome Biotechnologies, Aurora, CO, USA) was dissolved in dimethyl sulfoxide (DMSO) (Thermo Fisher Scientific, Waltham, MA, USA), and 3-MA (Tokyo Chemical Industry Co., Portland, OR, USA) was dissolved in Schneider's Drosophila Medium. Both small molecules were added into the cell culture directly [32, 33]. Wells of Aag-2 cells were assigned as the control, DMSO, rapamycin and 3-MA groups, with the latter three groups treated with DMSO, 50 nM rapamycin [21, 34] or 5 mM of 3-MA [21], respectively. Sextuplicate samples were examined for each treatment. For all treatment groups, cells were pretreated for 3 h and then infected with DENV-2 at a multiplicity of infection of 0.1 viruses per cell. After the virus was incubated with the cells for 1 h, the medium was discarded, and the cells were washed with phosphate-buffered saline (pH 7.4) before fresh medium was added. The infected cells were placed in the incubator for 2 days before being harvested for RNA and protein isolation.

### Western blot

The cells were lysed by RIPA buffer (25 mM Tris–HCl, 150 mM NaCl, 1% NP-40, 1% sodium deoxycholate and 0.1% sodium dodecyl sulfate [SDS]). The samples were incubated with RIPA buffer on ice for 15 min and then centrifuged at 13,400 *g* for 15 min. The protein concentration was measured using the BCA Protein Assay Kit (Thermo Fisher Scientific). The SDS protein sample buffer (MilliporeSigma, Burlington, MA, USA) was mixed with the 35 µg protein sample in a 4:1 ratio and then heated at 95 °C for 5 min. An SDS–polyacrylamide gel electrophoresis gel (10%) with running buffer (3.03 g Tris buffer, 14.41 g glycine, 1 g SDS in 1 l of ddH_2_O) was used to separate proteins by molecular mass. Proteins in the gel were transferred to nitrocellulose membranes (Invitrogen, Thermo Fisher Scientific, Carlsbad, CA, USA) using a transfer buffer (3 g Tris, 28.8 g glycine, 100 ml methanol in 1 l of ddH_2_O) in an Xcell SureLock Mini-Cell Electrophoresis Chamber (Thermo Fisher Scientific) maintained at 30 V for 1 h. The anti-LC3 antibody was kindly provided by Dr. Alexander Raikhel, Department of Entomology, University of California, Riverside (CA, USA) [35] and used as a primary antibody (1:1000), and anti-rabbit antibody (Abcam, Cambridge, MA, USA) was used as a secondary antibody (1:5000). β-Actin antibody (Cell Signaling Technology, Danvers, MA, USA) was used as the control (1:1000). Clarity Max Western ECL substrate (Bio-Rad Laboratories, Hercules, CA, USA) was applied to stimulate the enzyme horseradish peroxidase. The signal was detected on X-Omat film (Carestream, Rochester, NY, USA), and film development was completed with fixer and developer reagents (Kodak, Rochester, NY, USA). Each experiment was replicated to confirm that the western blot results were consistent.

### RNA extraction, reverse transcription and real-time PCR

TRIzol Reagent (Invitrogen, Thermo Fisher Scientific) was used to extract RNA from Aag-2 cells. Six samples were collected and RNA extraction was completed following well-established protocols [36, 37]; extracted RNA was stored at − 80 °C until use. The RNA samples were treated with RQ1 RNase-Free DNase (Promega, Madison, WI, USA). M-MLV Reverse Transcriptase (Promega) was used with oligo dT to form complementary DNA. Transcript levels were determined using the CFX96™ Real-Time PCR Detection System (Bio-Rad Laboratories). SsoAdvanced SYBR Green Supermix (Bio-Rad Laboratories) and specific primer sets designed for genes in the autophagy pathway were used to amplify gene products (Additional file 1: Table S1) [27]. The *Ae. aegypti* ribosomal protein S7 gene (GenBank Accession #AY380336) was used as a control for standardizing transcript levels [38]. For quantifying viral titer, quantitative real-time PCR, standardized with plaque assay, was completed with equal amounts of RNA from each sample (100 ng), the iTaq Universal SYBR Green One-step Kit (Bio-Rad Laboratories), DENV-2 specific primers (Additional file 1: Table S1), and following a standard protocol [39, 40]. The standard curves for DENV titer have been described previously [36], and the virus titer was presented as the mean of duplicates in log (base 10) plaque-forming unit equivalents per milliliter (log PFUe/ml) [37, 40].

### Statistical analysis

Gene expression data were analyzed by the Wilcoxon method in JMP Pro (www.jmp.com), to calculate the* P*-value and determine the presence of significant differences between each sample in gene expression studies. Holm-Šídák’s multiple comparisons test was used to calculate the *P*-value from the virus titer study. The figures were made through GraphPad Prism 9 (www.graphpad.com). The relative ratios of the proteins on the western blot were calculated using ImageJ (https://imagej.nih.gov/ij/) compared between LC3-II and β-actin.

## Results

### Activation of autophagy in Aag-2 cells after DENV infection

**Fig. 1:**
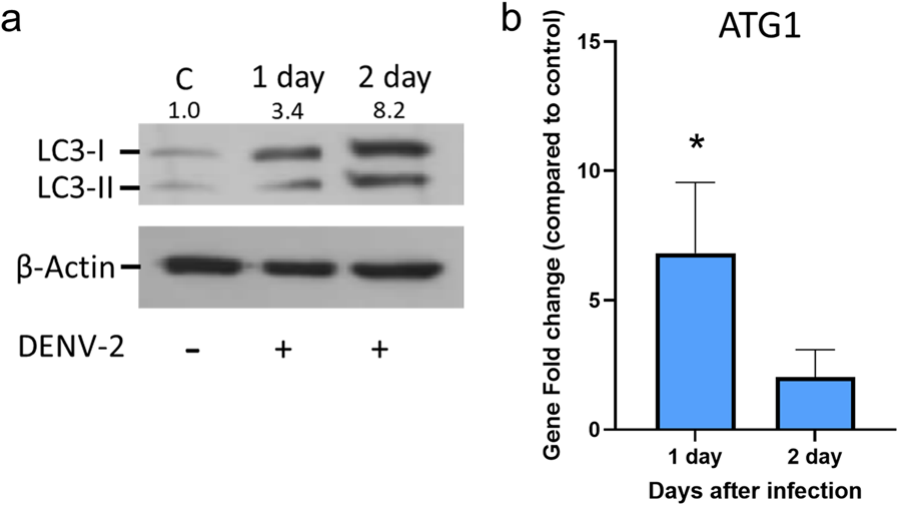
Evaluation of autophagy-related protein and transcript levels in Aag-2 cells after DENV infection. **a** Western blot results showing LC3-II protein level in Aag-2 cells after DENV infection. Aag-2 cells were infected with DENV and samples were collected after 1 and 2 days. The relative ratios of the density, calculated using ImageJ software, compared between the bands of LC3-II and β-actin are shown on the top of the blot. **b** Autophagy-related gene expression in Aag-2 cells at 1 and 2 days post-DENV infection by real-time PCR. Asterisk indicates a significant difference at *P* < 0.05 calculated by the Wilcoxon method. Abbreviations: ATG1 , Autophagy-related protein 1; C, control; DENV, dengue virus; LC3-I, microtubule-associated protein light chain 3; LC3-II, microtubule-associated protein light chain 3-phosphatidylethanolamine conjugate

Aag-2 cells were infected with DENV-2, and cell samples were collected on days 1 and 2 following DENV infection and analyzed for activation of the autophagy pathway. The western blot showed that the LC3-II protein level had increased on both day 1 and day 2 post-infection (Fig. 1a), which indicated activation of the autophagy pathway following DENV infection in Aag-2 cells.

Several ATG gene expression levels were checked after DENV infection in Aag-2 cells. ATG1 expression in the infection group was significantly increased after 1 day post-infection compared to the control group in the Aag-2 cells (Fig. 1b). However, ATG4, ATG5 and ATG12 transcripts did not differ after DENV infection. Differences in the levels of ATG gene expression, as well as in the levels of ATG1, did not occur after post-infection day 2 in DENV-infected Aag-2 cells.

### Manipulation of the autophagy pathway following exposure to small molecules and DENV infection in Aag-2 cells

**Fig. 2:**
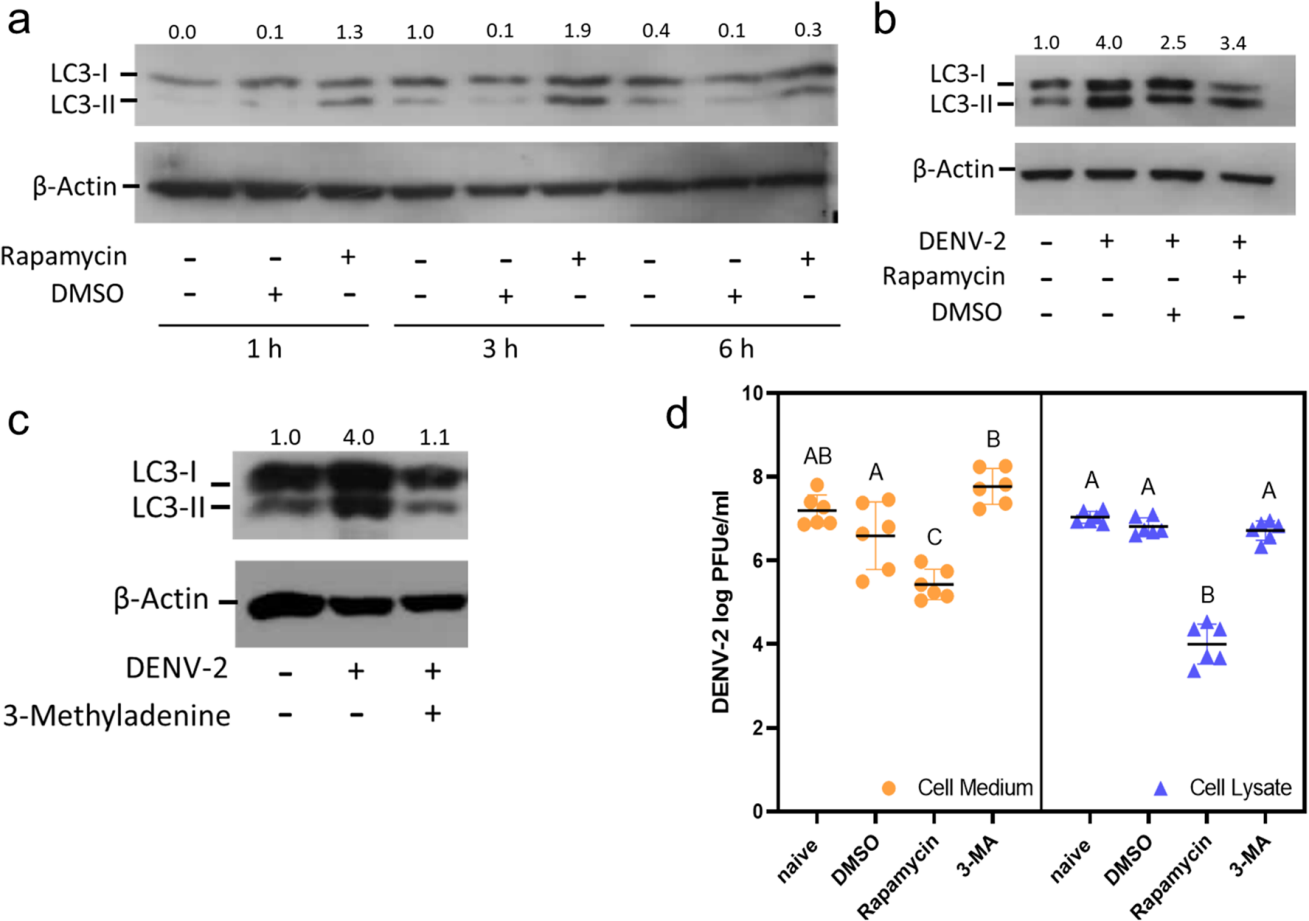
Manipulation of the autophagy pathway with small molecules and after infection with DENV in Aag-2 cells. **a** Western blot results showing LC3-II levels after treatment with rapamycin and/or DMSO for 1, 3 and 6 h. **b** Western blot results showing LC3-II levels 2 days after rapamycin, DMSO and/or DENV infection. **c** Western blot results showing LC3-II level 2 days after treatment with 3-MA or Schneider’s Drosophila Media and/or infected with DENV. The relative ratios of the density, calculated using ImageJ software, compared between the bands of LC3-II and β-actin are shown on the top of the blot **d** DENV log PFUe/ml titer changes in both Aag-2 cell lysate and cell medium pretreated with DMSO, rapamycin or 3-MA for 3 h before DENV infection for 2 days. Holm-Šídák's multiple comparisons test was used to calculate the *P*-value. Levels with the same uppercase letter are not significantly different. Abbreviations: DMSO, Dimethyl sulfoxide; 3-MA, 3-methyladenine; PFUe, plaque-forming unit equivalents

Rapamycin was used as an activator of autophagy in this experiment. Rapamycin (50 nM) was applied to cultured Aag-2 cells, and protein samples were collected after 1, 3, and 6 h post-exposure. An increase in LC3-II protein level was detected at 1 h following treatment, and LC3-II was stably expressed in both the 3- and 6-h post-exposure protein samples (Fig. 2a). Based on the LC3-II level results, 3 h of rapamycin pre-treatment was chosen to analyze the effects of DENV infection. The virus was incubated in Aag-2 cells for 2 days before cells were harvested. LC3-II was induced in the control, in cells pre-treated with DMSO and in cells pre-treated with rapamycin after DENV infection (Fig. 2b). However, no additional inducement of LC3-II conversion was noted in the pretreatment groups, indicating that the presence of both DENV and rapamycin might not have an additive effect on the formation of LC3-II.

3-MA was selected as the inhibitor of the autophagy pathway. After 3 h of 3-MA pre-treatment of cells, DENV was incubated with Aag-2 cells for 2 days. Previous western blots showed the activation of the autophagy pathway under DENV infection (Fig. 1a), but the LC3-II level decreased in the 3-MA treatment group (Fig. 2c). These results indicated that the autophagy pathway was inhibited by 3-MA in the Aag-2 cells.

After confirmation that both rapamycin and 3-MA function in the Aag-2 cells, the cells and culture medium were collected after 2 days of infection and the virus titer analyzed. The virus titer was 7.04 ± 0.14 log PFUe/ml in the cell lysate samples from the naïve group, 6.82 ± 0.20 log PFUe/ml in those from the DMSO treatment group, 4.00 ± 0.48 log PFUe/ml in those from the rapamycin treatment group and 6.72 ± 0.23 log PFUe/ml in those from the 3-MA treatment group The DENV titer between the naïve group, DMSO treatment group and 3-MA treatment group was not significantly different (naïve vs DMSO, *P* = 0.378; naïve vs 3-MA, *P* = 0.208; DMSO vs 3-MA, *P* = 0.566); however, the rapamycin-treated cell lysate had a significantly lower virus titer than the other groups (naïve vs rapamycin, *P* = 0.005; DMSO vs rapamycin, *P* = 0.005; 3-MA vs rapamycin, *P* = 0.005; Fig. 2d). DENV titer in the cell culture medium samples had a similar pattern as found in the cell lysates, with the naïve group having a titer of 7.20 ± 0.37 log PFUe/ml, the DMSO treatment group having a titer of 6.60 ± 0.81 log PFUe/ml, the rapamycin treatment group having a titer of 5.43 ± 0.36 log PFUe/ml and 3-MA treatment group having a titer of 7.77 ± 0.42 log PFUe/ml. The rapamycin-treated cell medium group contained significantly fewer virions than medium from the other three groups (naïve vs rapamycin, *P* = 0.005; DMSO vs rapamycin, *P* = 0.02; 3-MA vs rapamycin, *P* = 0.005). There was no difference between the cell medium titer of the naïve group and the DMSO treatment group (naïve vs DMSO, *P* = 0.12). Although the 3-MA treatment group had a higher titer, no significant difference between naïve and 3-MA groups was noted (Fig. 2d).

### Autophagy-related gene expression level differences following exposure to small molecules and DENV infection in Aag-2 cells

**Fig. 3:**
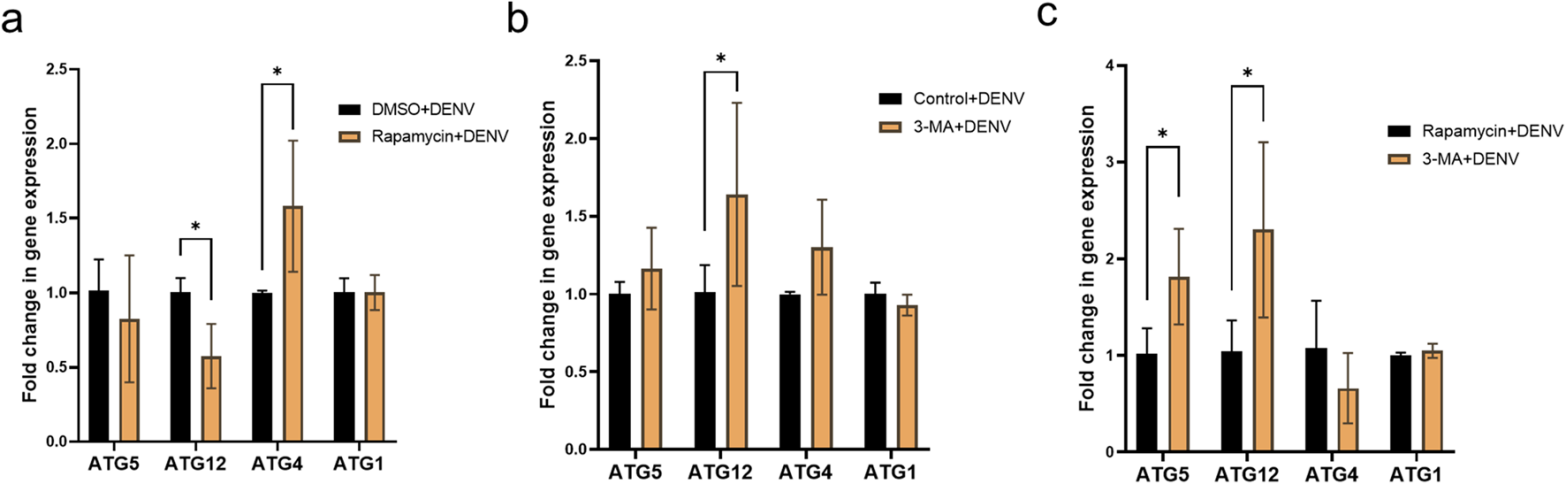
Autophagy-related gene expression level following treatment with the small molecules and infection with DENV in Aag-2 cells for 2 days. **a** Fold change in ATG transcript levels in cell lysates after pretreatment with rapamycin and DMSO and infected with DENV. **b** ATG transcript level differences in cell lysates after pretreatment with 3-MA and infected with DENV. **c** ATG transcript level altered between rapamycin and 3-MA treatment under DENV infection. Transcript level differences between control and treatment groups were analyzed using the Wilcoxon method. Asterisk indicates a significant difference between treatment groups at *P* < 0.05 calculated using the Wilcoxon method

Gene transcription of four ATG genes was checked after treatment with the small molecules and DENV infection in Aag-2 cells. After 2 days of infection, ATG gene expression in the rapamycin treatment group was compared to that in the DMSO treatment group, and ATG gene expression in the 3-MA treatment group was compared to that in the control group. In the rapamycin treatment experiment, the fold change ATG12 expression was 0.58 ± 0.21 and was significantly lower than that in the DMSO treatment group (*P* = 0.03). Transcription of ATG4 showed a 1.58 ± 0.44-fold change in expression, which was significantly higher than that in the DMSO group (*P* = 0.02). Both ATG1 and ATG5 did not show any transcript level differences between the rapamycin-treated cells and the DMSO-treated cells when cells were infected with DENV (Fig. 3a). Under the 3-MA treatment, DENV infection in the Aag-2 cells did not alter the transcript levels of ATG1, ATG4 and ATG5. However, ATG12 was detected to have a significantly higher fold change in expression (1.64 ± 0.59; *P* = 0.04) compared to the control (Fig. 3b).

To evaluate the expression level of ATG between the two groups treated with the small molecules under DENV infection, we compared the relative gene fold change (Fig. 3c). The transcript levels of both ATG5 and ATG12 were significantly higher in the 3-MA group than in the rapamycin group (ATG5: 1.81 ± 0.49, *P*-value = 0.04; ATG12: 2.30 ± 0.91, *P* = 0.02). There were no differences in the expression levels of ATG4 and ATG1 between the two small-molecule treatment groups.

## Discussion

Autophagy as a regulated cellular degradative pathway is induced by several factors and is associated with DENV infections in humans. DENV infection triggers the activation of the autophagy pathway and promotes virus replication [21]; however, the interaction between autophagy and DENV in the mosquito is not clear. Here we provide evidence that activation of the autophagy pathway could interfere with DENV infection and serve as an antiviral mechanism in an *Ae. aegypti* cell line. Moreover, the results provide a piece of fundamental information needed to proceed with future experiments and to improve understanding of the role of autophagy in *Ae. aegypti*.

The application of the LC3 antibody to detect protein levels of LC3-II is one of the common ways to monitor the activation of autophagy [12, 13]. LC3-II activation by DENV has been shown in several mammalian cell lines, including Huh7 [21], HepG2 [41] and MDCK [22] cell lines. Here we showed the LC3-II protein level increased in cells of the* Ae. aegypti*-derived cell line Aag2 after DENV infection (Fig. 1a), which is indicative that the autophagy pathway may be associated with the virus in mosquitoes.

Small molecules, such as rapamycin and 3-MA, have been widely used to manipulate the autophagy pathway and to gain an understanding of the interaction with DENV [20]; moreover, both rapamycin and 3-MA have proven useful in studying pathway function in mosquito systems [42, 43]. Therefore, we utilized these molecules in our study and showed their impacts on the autophagy pathway (Fig. 2a–c). In the mammalian system, studies have established that DENV activates autophagy upon infection, and the virus is suspected to use the autophagic process as part of its replication strategy in liver cells [21, 23, 41]. Surprisingly, in the treated Aag-2 cells, activation of the autophagy pathway reduced the virus titer while inhibition of the autophagy pathway slightly increased the DENV titer (Fig. 2d), suggesting that the autophagy pathway is a possible antiviral mechanism in the mosquito system. Activation of the autophagy pathway in Aag-2 cells resulted in a significant decrease in virus titer, similar to that previously reported in a mammal monocytic cell study [44]. Moreover, the autophagy pathway was activated through Toll-7 in *Drosophila *[45] infected with vesicular stomatitis virus [18]. Although inhibition of the autophagy pathway through exposure to 3-MA did not significantly increase the virus titer in our study (Fig. 2d), bafilomycin A1 and spautin-1, two chemicals known to also serve as inhibitors of the autophagy pathway, promoted viral replication in Aag-2 cells in a previous study [46]. This absence of a significant change in viral titer with 3-MA treatment may imply that the autophagy pathway serves as a minor antiviral mechanism in the mosquito system. Additionally, studies have also suggested that autophagy does not play a major role in antiviral immunity in *Drosophila *[47]. However, more studies are needed to rule out participation of the autophagy pathway in antiviral processes.

In the present study, the autophagy pathway was manipulated using both rapamycin and 3-MA in Aag-2 cells. Although the influence of these small molecules on other pathways is a possibility, our aim was to assess their effects on autophagy and DENV titer. Rapamycin affected the activity of downstream S6 kinase and eukaryotic initiation factor 4E-binding protein-1 related to cell growth in mammals [48], but the effects of rapamycin treatment have not been sufficiently studied in mosquitoes. When mosquitoes were exposed to rapamycin, amino acid-based TOR signaling was inhibited in *Ae. aegypti* [42], while this treatment enhanced activation of immune responses in *Anopheles stephensi* [49]. A previous study used torin-1, another mTORC inhibitor, to investigate the interaction between virus and mosquito. The authors found that the treatment did not induce LC3-II protein [46], suggesting that rapamycin could be a better small molecule to investigate autophagy activation in the mosquito. On the other hand, 3-MA has been noted to regulate the inflammatory response in mammals [50]. Future studies should investigate the likely effects of other pathways under small molecule treatment and their potential influences on DENV infection.

We analyzed the expression of several ATG genes shown previously to be upregulated after blood-feeding [27], but in our study only the ATG1 gene transcript level was significantly increased after DENV infection in the Aag-2 cells and no difference was detected in the other three genes (Fig. 1b). These results suggest that the autophagy-related transcriptomic changes may not be significantly altered after DENV infection. Interestingly, the transcription levels of ATG12 were altered under small molecule treatment and after DENV infection in the Aag-2 cells (Fig. 3). Specifically, ATG12 expression was increased in the 3-MA and DENV treatment groups only (Fig. 3b), but there were no effects on DENV titer compared to the control group. Rapamycin and DENV treatment resulted in lower ATG12 gene expression, but the DENV titer was lower compared to the DMSO treatment (Fig. 3a). Taken together, these findings suggest that ATG12 may be involved in DENV replication. Treatment with rapamycin and DENV infection also resulted in increased ATG4 expression, suggesting an additional role for ATG4 in DENV infection. Both ATG4 and ATG12 are involved in autophagosome formation and given their importance in the autophagy pathway [4], these genes could be potential target genes to investigate for interference with DENV infection or replication in the mosquito. Additionally, Aag-2 cells treated with 3-MA had higher transcript levels of ATG5 and ATG12 compared to the rapamycin group under DENV infection (Fig. 3c). One possibility is that the autophagy pathway could have negative feedback that suppresses ATG gene transcription, thus blocking pathway activation [51]. Although the regulation between the protein and messenger RNA level in the autophagy pathway has not been well studied, here we provide evidence that inhibition of the autophagy pathway enhanced ATG gene transcription and vice versa.

## Conclusions

In summary, we demonstrated the interaction between DENV and the autophagy pathway in mosquito cells*.* The results also reveal that the autophagy pathway plays a role in the antiviral response in the mosquito. Given the importance of the autophagy pathway in mosquito physiology, such as egg maturation [35], it is crucial to investigate the influence of autophagy in the mosquito and perhaps in different mosquito tissues. Moreover, evaluating the usefulness of the autophagy pathway by gene silencing or small molecules to interfere with virus transmission would validate the role of this pathway in mosquito-borne virus infection. Together, our results show the practicality of small molecules in altering the autophagy pathway, and the impact on the DENV infection, indicating that the use of small molecules as possible mosquito pathogen vaccines should be evaluated.

## Supplementary Information

**Additional file 1: Table S1.** Primer list for real-time PCR.

## Data Availability

No new data were created or analyzed in this study. Data sharing is not applicable to this article.
