# Supplementary material for: Activation of the autophagy pathway decreases dengue virus infection in Aedes aegypti cells
Source: Parasit Vectors. 2021 Oct 26;14:551. doi: 10.1186/s13071-021-05066-w (PMC8549150; doi:10.1186/s13071-021-05066-w)
Supplement: Supplementary file 1 — Additional file 1: Table S1. Primer list for real-time PCR. [file 13071_2021_5066_MOESM1_ESM.pdf]

Table S1. Primer list for Real-time PCR

Gene	Gene ID	Sequence (5'→3')
ATG1 F	AAEL016987	GCGATACGAATGTGCCAAAC
ATG1 R	AAEL016987	GTGCCAGTGAATGGAGAAGA
ATG5 F	AAEL002286	CTCAGCTATCCGGACAACTTTC
ATG5 R	AAEL002286	GAACCGTCATCTCAAGCCTTAC
ATG4 F	AAEL010516	GTCGCCTTCGGAGATATTTGT
ATG4 R	AAEL010516	AGCTTCGATGTCCTCCATTTC
ATG12 F	AAEL009089	ATTCTCCATGCAACGGGTAG
ATG12 R	AAEL009089	TATCTGATCTGGCGATGGTG
S7 F	AY380336	ACAAGAACCAGCAGACCAC
S7 R	AY380336	TCCGGGAATTCGAACGTAAC
DENV-2 F	KM204118	CTWTCAATATGCTGAAACGCG
DENV-2 R	KM204118	CGCCACACAAGGGCCATGAACAG
